# New Instructions for Authors of Reports Published in *MMWR* Weekly

**Published:** 2014-01-10

The *MMWR* Weekly policy regarding listing of authors has changed. Contributing authors are now listed as “authors,” rather than “contributors.” Criteria for authorship of all *MMWR* reports now follow the recommendations of the International Committee on Medical Journal Editors (ICMJE): “Authorship credit should be based on 1) substantial contributions to conception and design, acquisition of data, or analysis and interpretation of data; 2) drafting the article or revising it critically for important intellectual content; and 3) final approval of the version to be published. Authors should meet conditions 1, 2, and 3” (available at http://www.icmje.org/ethical_1author.html).

In accordance with CDC policy, the order for listing authors should be a joint decision of the coauthors. *MMWR* recommends that author order be discussed early during collaboration and revised as needed as the work progresses, and that authorship order, including choice of first author, should be based on the level of contribution to the report and the work underlying it. The first author will have responsibility for the integrity of the work as a whole from inception to publication. The requirement that all *MMWR* reports be cleared at CDC before publication is unchanged, and content remains in the public domain. *MMWR* instructions for authors have been updated and are available at http://www.cdc.gov/mmwr/contributors/index.html.

